# Borreliosis and doxycycline treatment disrupt gut microbiota and immune responses in nonhuman primates

**DOI:** 10.1128/mbio.01437-25

**Published:** 2025-06-27

**Authors:** Ethan G. Napier, Isaac R. Cinco, Sheridan B. Wagner, Ethan V. Stuart, Qi Qiao, Joshua Taylor, Brian Stevenson, Ilhem Messaoudi

**Affiliations:** 1 Department of Microbiology, Immunology, and Molecular Genetics, College of Medicine, University of Kentucky 12252, Lexington, Kentucky, USA; 2 Oregon National Primate Research Center, Oregon Health & Science University 88960, Beaverton, Oregon, USA; Georgia Institute of Technology, Atlanta, Georgia, USA

**Keywords:** *Borrelia burgdorferi*, microbiome, doxycycline, single-cell RNA sequencing, macaque

## Abstract

*Borrelia burgdorferi* (Bb) is the spirochete that causes Lyme disease (LD). Transmission to humans occurs via bites of infected *Ixodes* spp. ticks. Doxycycline is often used to treat LD patients, which is sometimes delayed due to current diagnostic challenges. Recently, several lines of evidence show dysbiosis of the gut microbiome in LD patients, which may be a host factor contributing to pathogenesis and symptomology but is still an understudied area in the field. To address this knowledge gap, we longitudinally compared the immune response and gut microbial composition of Japanese macaques after intradermal infection with Bb strain 297 with or without doxycycline treatment. Immunological and microbial changes were monitored longitudinally using enzyme-linked immunosorbent assay, flow cytometry, single-cell genomics, and 16S amplicon sequencing assays. All Bb-inoculated animals seroconverted to produce Bb-specific antibodies. Borrelial DNA was undetected by real-time quantitative PCR in the skin, blood, and cerebrospinal fluid. Levels of CXCL13, a B cell chemoattractant, increased following infection but reached a nadir after treatment. The frequency of circulating immune cells remained constant over the course of the study, but gene expression analysis showed downregulation of antimicrobial genes in monocytes, dendritic cells, and B cells until late in infection, regardless of treatment. Infection and antibiotic treatment led to a loss of gut commensals important for maintaining gut integrity. Finally, untreated infected animals showed mononuclear myocarditis along with sporadic detection of borrelial DNA. Data from this study suggest aberrant B cell activity, myocarditis, and loss of important commensal microbes as factors contributing to symptoms of untreated Lyme disease.

**IMPORTANCE:**

Lyme disease (LD) is caused by *Borrelia burgdorferi* (Bb) transmitted via tick bite. The incidence of LD is expanding in North America and Southeast Asia. LD patients are frequently misdiagnosed or receive delayed treatment due to the lack of sensitive diagnostic strategies. The pathophysiology of LD remains poorly understood because of challenges with clear infection timelines in clinical studies. Here, we utilize Japanese macaques to provide an in-depth longitudinal investigation into the host immunological and gut microbial changes in response to Bb infection. This work highlights CXCL13 as a potential Bb diagnostic marker, as well as host factors such as aberrant B cell activity, mononuclear myocarditis, and gut dysbiosis as potential therapeutic targets.

## INTRODUCTION

Lyme borreliosis or Lyme disease (LD) is a tick-borne disease caused by *Borrelia burgdorferi* (Bb), a spirochete transmitted to mammals by *Ixodes scapularis* ticks. LD is the most common vector-borne disease in the United States with nearly 500,000 new cases annually (1). Early localized LD (stage 1) is characterized by the erythema migrans rash that is localized to the skin before the onset of systemic symptoms or dissemination (2, 3). Early disseminated LD (stage 2) cases occur days to weeks after a tick bite and are characterized by non-localized reactions, such as satellite rashes, carditis, and acute neurologic disease. Finally, late disseminated disease (stage 3) occurs months to years later and typically includes Lyme arthritis, acrodermatitis atrophicans, polyneuropathy, and encephalopathy (4). Clinicians often rely on non-specific symptoms such as low-grade fever, myalgias, arthralgias, or headache for diagnosis (4, 5), as many common diagnostic strategies are inadequate, as the bacteria are difficult to detect by PCR amplification (6–8). For instance, serology is an excellent marker for mid- to late-stage LD, but Bb-specific IgM or IgG titers are too low in early stages for diagnosis (9–13). This presents a major challenge for initiating antibiotic treatment within the optimal timeframe. It is important to initiate treatment early as Bb have several immune evasion strategies, notably antigenic variation preventing complement and adaptive immune system recognition (14–17). The mainstream treatment for LD, doxycycline, may contribute to LD symptoms due to its disruption of the gut microbiome (18, 19). Indeed, gut dysbiosis has been implicated in the development and exacerbation of chronic fatigue syndrome (20), inflammatory arthritis (21), and a variety of neurologic disorders (22), which are all common symptoms among LD patients.

Therefore, in this study, we aim to better understand how borreliosis, along with doxycycline treatment, modulates the systemic immune response and affects the gut microbiome. These key questions are difficult to investigate in clinical studies due to undefined infection timelines and other confounding variables such as diet, co-morbidities, medications, and substance use. Therefore, several animal models of LD have been developed. Rodents have inherent limitations in studying human LD as they are closely related to Bb reservoirs such as the white-footed mouse (*Peromyscus leucopus*) (23), and their immune system and gut microbial communities are too dissimilar compared to humans. In contrast, the macaque model of borreliosis closely recapitulates several hallmarks of human disease, such as the EM rash with perivascular lymphocytic infiltrations, conjunctivitis, and Bb-specific IgM and IgG production (24–26). In addition, nonhuman primates share close physiological similarities to humans and overcome confounders that often complicate clinical studies.

**Fig 1:**
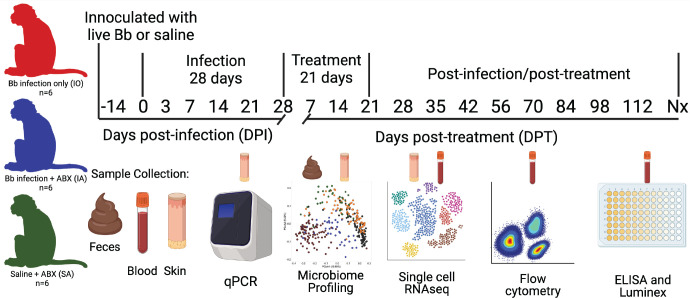
Project design. Japanese macaques were distributed into three groups of six animals. Twelve animals were inoculated intradermally with a total dose of 10^4^ live Bb strain 297 at six individual sites on the thoracic dorsum to mimic natural tick-bite infection. Six animals received an equal volume of saline injections. Doxycycline (4 mg/kg of body weight/day) was administered to six infected animals (infection + antibiotics, IA) and six uninfected animals (saline injection + antibiotics, SA) at 28 or 42 days post-infection (DPI) and lasted for 21 days. The other six infected animals went untreated for the entire study (infection only, IO). Fecal and blood samples were collected at the indicated DPI or days post-treatment. Skin biopsies at the injection sites were collected at −14, 3, and 7 DPI. Peripheral blood mononuclear cells were probed via scRNA-seq, flow cytometry, ELISA, and Luminex assays. Microbiome profiling was performed on fecal swabs. Bulk RNA sequencing was performed on the skin biopsies. Data from IO animals (IO1–IO6) are plotted in red, IA animals (IA1–IA6) are plotted in blue, and SA animals (SA1–SA6) are plotted in green.

Here, we used Japanese macaques to study longitudinal changes in the immune response and gut microbiota after Bb infection and subsequent doxycycline treatment. Three experimental groups were studied to examine the effects of infection only (IO), infection plus antibiotic treatment (IA), and saline injection plus antibiotic treatment (SA) (Fig. 1). We employed a systems biology approach combining DNA and RNA sequencing, flow cytometry, enzyme-linked immunosorbent assay (ELISA), Luminex, and histology. Collectively, our data show that macaques have a delayed immune response to Bb, develop gut microbial dysbiosis, and exhibit mononuclear myocarditis. Thus, LD pathogenesis may be associated with loss of short-chain fatty acid (SCFA)-producing bacteria, aberrant B cell activity, and deposition of immune cells into heart tissue.

## RESULTS

### Bb infection remodels the transcriptome at the injection site

We first analyzed the injection site at 3 and 7 days post-infection (DPI). Interestingly, we were unable to detect Bb DNA at any of the time points analyzed (data not shown). We were also unable to detect Bb following inoculation of culture media with skin explants (data not shown). These data could be explained by the ability to quickly migrate from the site of infection (27, 28) or due to the low inoculum of 10^4^ spirochetes distributed across six sites. To uncover transcriptional changes induced by infection (Fig. 1), bulk RNA sequencing of skin biopsies obtained before and 3 and 7 days post-infection was carried out. Analysis of differentially expressed genes (DEGs) at 3 and 7 DPI relative to pre-infection was carried out for each group because of the outbred nature of the animals and the need to compare transcriptional changes within each animal rather than between groups. Principal component analysis showed evolution of the skin transcriptome between pre-infection and 7 DPI (Fig. S1A).

In the saline group, there was minimal DEG overlap between 3 and 7 DPI relative to the pre-infection time point, with only seven shared DEGs that enriched to leukocyte migration and response to interleukin-1 (*CXCL8* and *CCL20*) gene ontology (GO) terms (Fig. S1B and C). DEGs at 3 DPI enriched to processes associated with tissue repair (e.g., epidermis development and establishment of skin barrier; *KRT, LCE*) and immune responses (e.g., immune effector process, humoral immune response; *IL6, ICAM1,* and *IGHM*) (Fig. S1B and C). At 7 DPI, DEGs mapped exclusively to the GO terms related to tissue repair, such as extracellular matrix structural constituent and epidermis development (*COL* and *KRT* genes) (Fig. S1B and C).

The infected animals exhibited more DEGs in response to infection compared to saline injection. A larger overlap in DEGs was noted between the two time points in the infection group with 74 DEGs that enriched to processes involved in wound repair (*HBEGF* and *MT2A*) as well as immune responses to pathogens (*CCL20, IL6,* and *SLAMF1*) (Fig. S1D and E). DEGs at 3 DPI were enriched in the regulation of inflammatory response and response to bacterium (*CXCL8, S100A8,* and *GPR4*), while those at 7 DPI were uniquely enriched in humoral immunity and inflammatory processes (*TNFSF9* and *NOD2*) (Fig. S1D and E).

**Fig 2:**
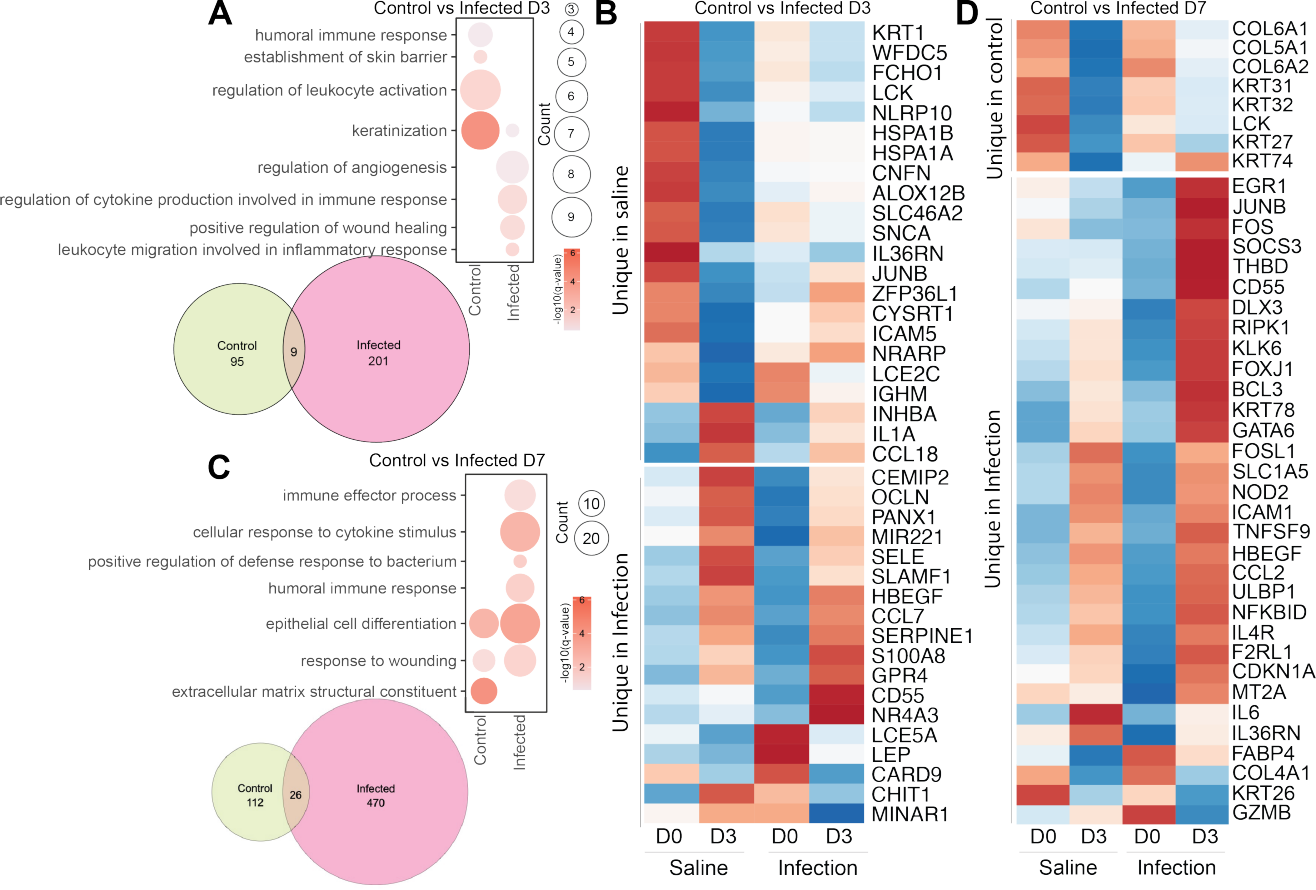
Skin at the injection site is undergoing repair and antimicrobial processes. Bulk RNA sequencing of skin biopsies at the inoculation site. Analysis comparing transcriptional changes was done for (**A and B**) infection and SA animals at 3 DPI and (**C and D**) infection and SA animals at 7 DPI.

Next, we compared DEGs detected 3 and 7 DPI in the saline-injected control (SA) and infected animals (Fig. 2). A very small number of DEGs was shared between the two conditions, with only nine shared DEGs at 3 DPI and 26 shared DEGs at 7 DPI indicative of different responses. In line with the results described above, DEGs unique to the SA group at 3 DPI were enriched in skin barrier repair (*KRT1* and *LCE2C*) and immune activation (*IL1A* and *IGHM*), while those unique to the infection condition enriched in inflammatory processes (*SERPINE1* and *CD55*) and immune responses (*S100A8* and *SERPINE1*) (Fig. 2A and B). Similarly, at 7 DPI, DEGs uniquely detected in the SA group exclusively mapped to barrier repair (*KRT* and *COL* genes), while those in inoculated animals mapped extensively to host defense and inflammation (*FOS, IL6,* and *CCL2*), as well as tissue repair (*HBEGF* and *MT2A*) (Fig. 2C and D).

### Bb infection dysregulates antibody and systemic inflammatory responses

**Fig 3:**
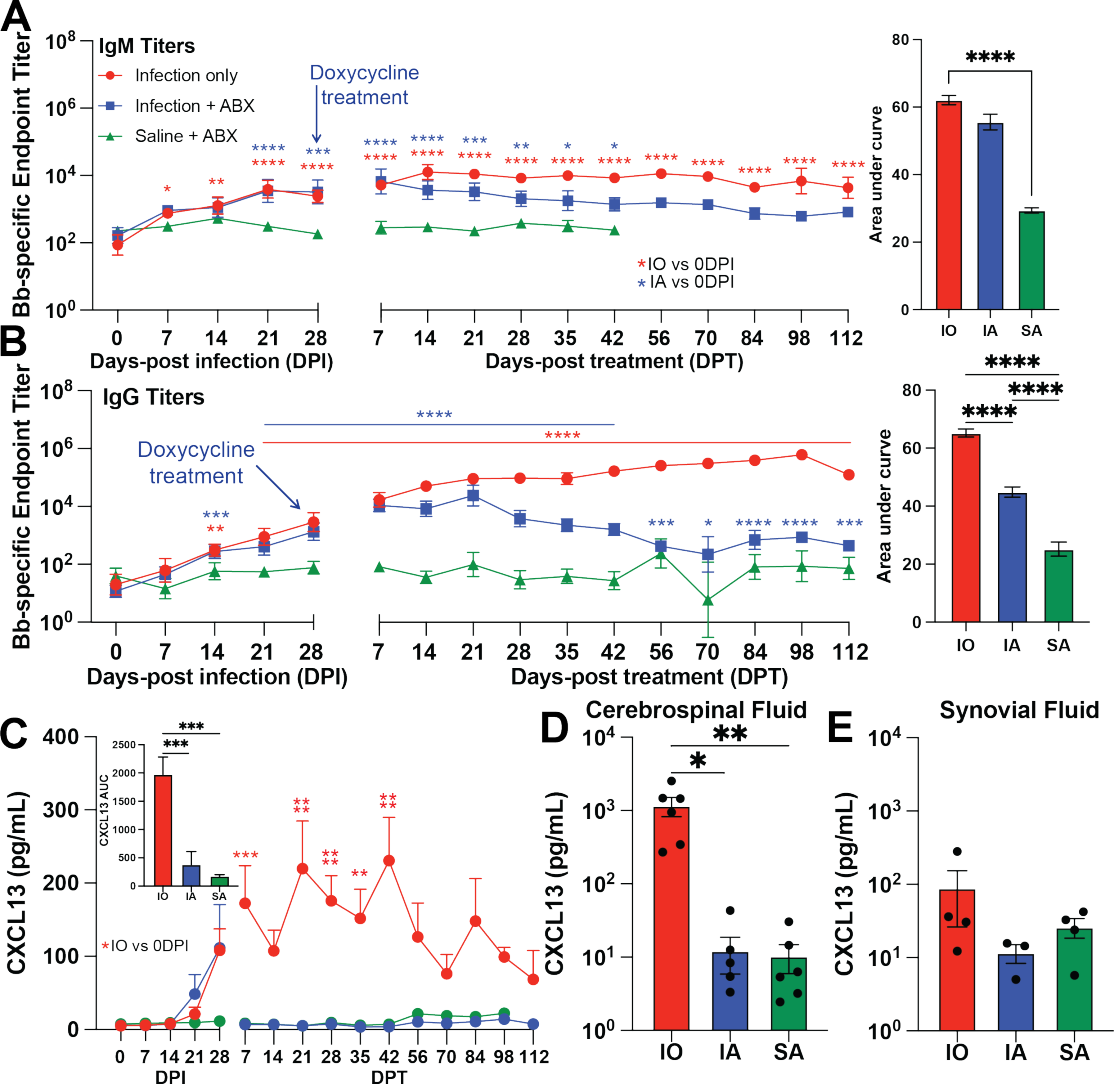
Doxycycline reduces immunoglobulin secretion and CXCL13 production in infected animals. Bb-specific (**A**) IgM and (**B**) IgG endpoint titers and area under the curves over the course of infection, as determined by ELISA with whole cell extract of Bb strain 297. (**C**) Plasma CXCL13 abundance over the course of infection and the area under the curve analysis. Red * indicates a significant increase in the IO group at that time point compared to 0 DPI. Blue * indicates a significant increase in the IA group at that time point compared to 0 DPI. (**D**) Cerebrospinal fluid and (**E**) synovial fluid CXCL13 concentrations (in arbitrary units) at necropsy.

Bb-specific IgM and IgG titers were detected in infected animals at 7 and 14 DPI, respectively. While IgM levels remained detectable in the IO group throughout the entire study, they declined post-doxycycline treatment (DPT) in the infected animals that received antibiotics (IA) (Fig. 3A). Similarly, IgG levels were first detected 14 DPI and remained detectable throughout the study in infected animals but started declining 35 days after doxycycline treatment. IgG levels were higher in IO compared to SA animals starting at 21 DPI. Overall, IgM levels were significantly higher only in the IO group compared to the SA group. On the other hand, IO animals produced the most IgG, followed by IA animals, as indicated by the area under the curve calculations (Fig. 3B).

We then assessed longitudinal changes in cytokine, chemokine, and growth factor levels in plasma by Luminex. No significant changes in any of the mediators measured were induced by infection, except for CXCL13, which increased in the infected animals at 28 DPI (Fig. 3C). While levels of CXCL13 remained elevated in the IO group, they dropped to baseline in the IA group 1 week after the initiation of doxycycline treatment (Fig. 3C). CXCL13 is a B cell chemoattractant, which has been identified as a potential biomarker for Lyme neuroborreliosis in cerebrospinal fluid (CSF) (29). Therefore, we performed a CXCL13 ELISA on CSF and synovial fluid collected at necropsy. CXCL13 levels were elevated in the CSF of IO animals compared to those of IA and SA animals (Fig. 3D), but no significant differences were noted in the synovial fluid (Fig. 3E).

The increased levels of this B cell chemoattractant led us to perform an in-depth analysis of immune cell frequency in peripheral blood by flow cytometry (Fig. S2A and B). Although the relative abundance of B cells was unchanged (Fig. S2C), we observed modest increases in the frequency of proliferating naive and marginal zone B cells in the infected groups compared to the SA group at 28 DPI, 7 DPT, and 28–35 DPT (Fig. S2D and E). Frequency of other immune cells (dendritic cells, NK cells, and T cells) was mostly unchanged, indicating a lack of systemic response (Fig. S2F through H).

### Bb infection disrupts the antimicrobial transcriptional program within monocytes, DCs, and B cells

**Fig 4:**
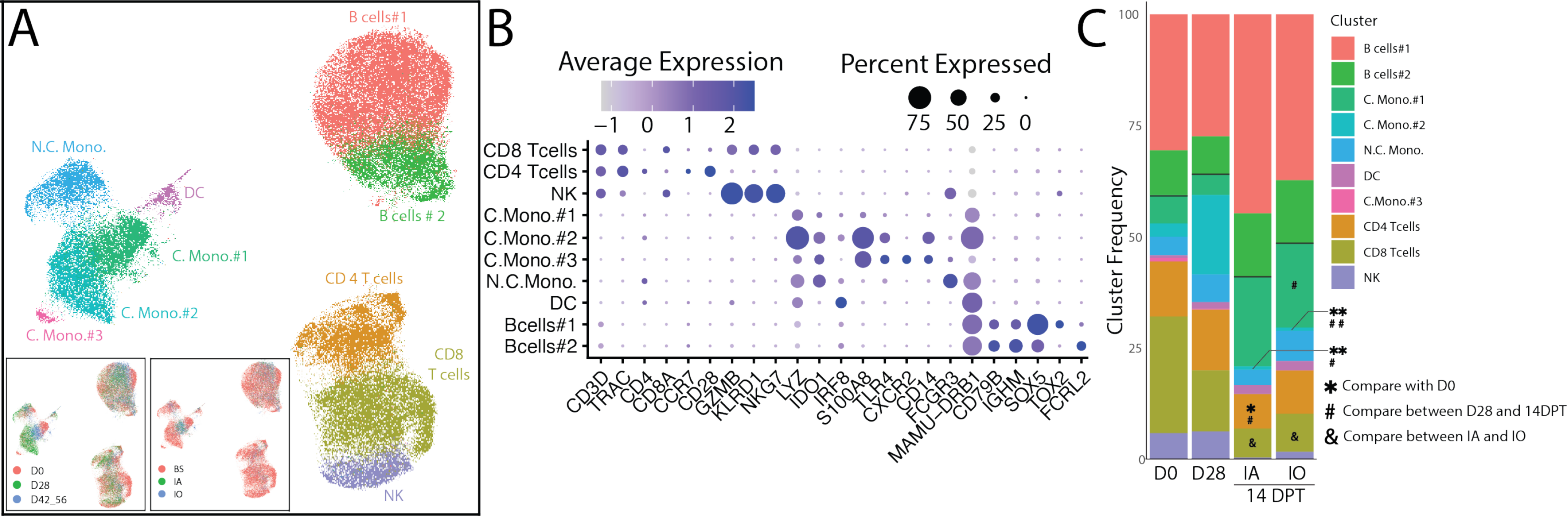
Single-cell RNA sequencing analysis reveals differences in monocyte and T cell subsets. (**A**) Uniform manifold approximation and projection representation of immune cells within three timepoints (0 and 28 DPI, 14 DPT) across IA and IO groups showing 10 unique clusters. (**B**) Bubble plot of key gene markers used to annotate the UMAP. (**C**) Frequency of clusters by DPI/DPT. * indicates significance between 0 DPI and 14 DPT, # indicates significance between 28 DPI and 14 DPT, and & indicates significance between IA and IO.

Although we did not detect changes in immune cell frequencies, we used single-cell RNA sequencing to determine immune responses to Bb infection at the transcriptional level. We interrogated peripheral immune cells of infected animals at 0 and 28 DPI, as well as at 14 DPT, to capture transcriptional changes induced by Bb infection before doxycycline treatment, as well as after 14 days of treatment. T cells were identified based on *CD3* and *TRAC* expression, then delineated into CD8 (*CD8A, GZMB, KLRD1,* and *NKG7*) and CD4 (*CD4* and *CD28*) T cells (Fig. 4A and B). NK cells had increased expression of *GZMB, KLRD1,* and *NKG7* compared to CD8 T cells. Classical monocytes were classified by the expression of *LYZ, IDO1, S100A8, TLR4, CD14,* and *MAMU-DRB1*. Non-classical monocytes expressed higher levels of *FCGR3* compared to classical monocytes. DCs were identified by high *IRF8* expression. B cells uniquely expressed *CD79B* and *IGHM*. The two B cell populations were identified to have differential expression levels of *SOX5*, *TOX2*, and *FCRL2* (Fig. 4B).

**Fig 5:**
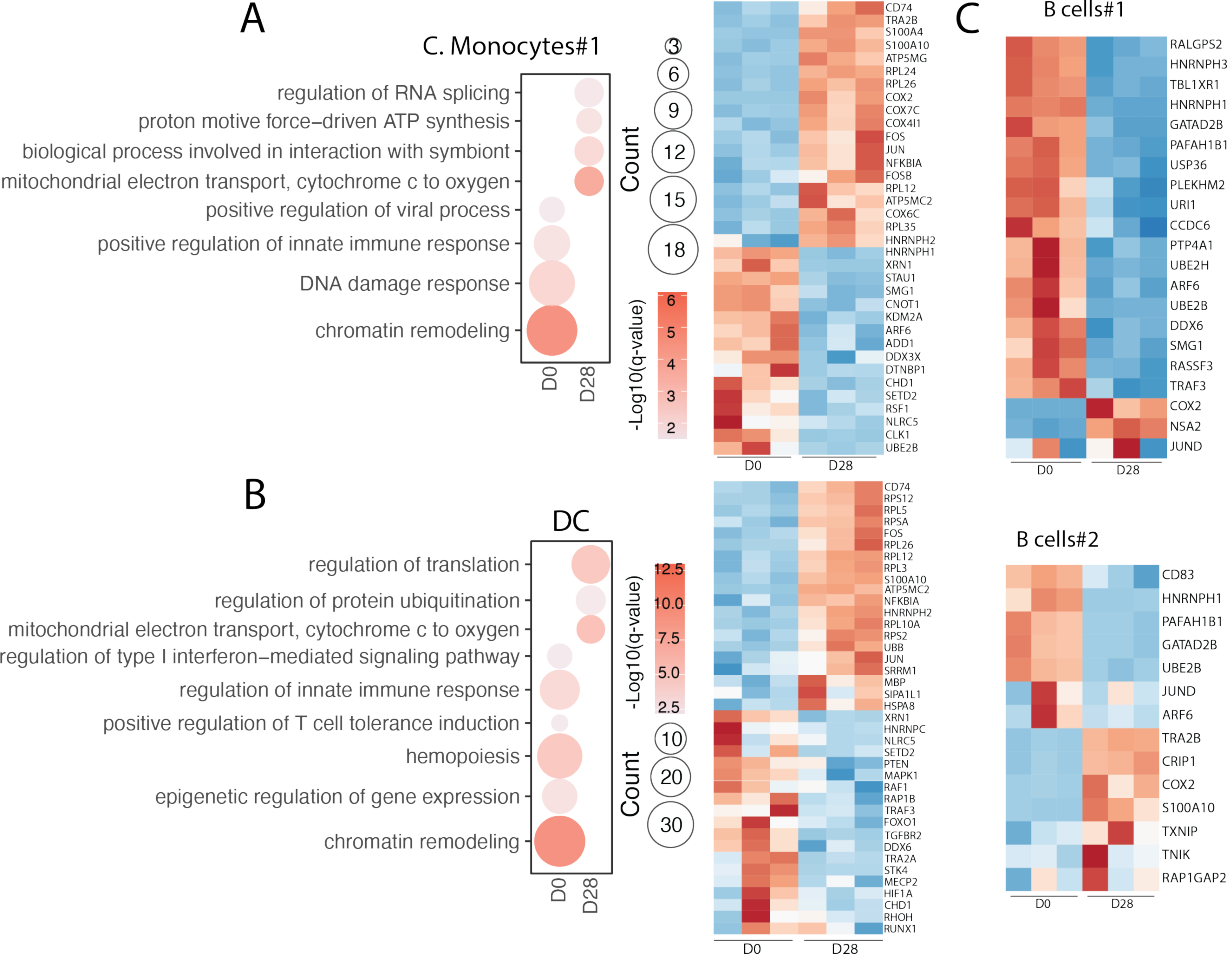
Monocytes, DCs, and B cells have anti-inflammatory shifts in the peripheral immune landscape. (**A**) Bubble plot of the GO terms to which DEGs within monocyte cluster #1 from IO and IA animals enriched at 0 and 28 DPI, along with the heatmap of DEGs. The size of the bubble indicates the number of DEGs that are enriched in its respective GO term, and the −log(*q*-value) is indicated by color. Scaled average gene expression is represented by the color within each heatmap from low (blue) to high (red). (**B**) Bubble plots of the gene ontology terms to which DEGs within the dendritic cell cluster from IO and IA animals enriched at 0 and 28 DPI, along with the heatmap of DEGs. (**C**) Heatmaps of significantly differentially expressed genes within B cell clusters #1 and #2 of IO and IA animals at 0 and 28 DPI.

No significant changes in the relative abundance of any of the clusters were detected at 28 DPI. Moreover, our analysis revealed no major transcriptional changes between 0 and 28 DPI except within classical monocytes #1 and DCs (Table S1; Fig. 5). The expression of genes associated with RNA splicing (*HNRNPH2*), mitochondrial energy production (*ATP5MC2* and *COX4I1*), and interactions with symbiont (*CD74* and *S100A4*) was increased at 28 DPI in monocytes. On the other hand, the expression of genes involved in cellular growth and immunity was downregulated (*SETD2, XRN1, RSF1,* and *NLRC5*) (Fig. 5A). Similarly, upregulated DEGs within DCs played a role in mRNA and protein degradation (*RPSA, HNRNPH2,* and *UBB*) and mitochondrial metabolism (*ATP5MC2*), while downregulated DEGs are important for inflammatory processes (*TRAF3, HIF1A, NLRC5,* and *MAPK1*) (Fig. 5B).

We also examined transcriptional changes within B cells due to the marked increases in CXCL13 and dysregulated antibody responses. Expression of genes important for mRNA metabolism (*HNRNPH1, SMG1,* and *DDX6*) as well as protein stability (*UBE2B* and *USP36*) was decreased at 28 DPI in B cell subset #1 (Fig. 5C). Similarly, reduced expression of genes involved in cellular activation (*CD83, JUND,* and *ARF6*), mRNA processing (*HNRNPH1*), and epigenetic regulation (*GATAD2B*) was also detected in the second B cell cluster. On the other hand, genes that play a role in host defense (*COX2, NSA2,* and *JUND*) were upregulated in subset #1, and genes important for differentiation and survival (*TNIK, RAP1GAP2,* and *S100A10*) were upregulated in subset #2 (Fig. 5C).

### Doxycycline treatment limits the inflammatory response in monocytes, DCs, and B cells

**Fig 6:**
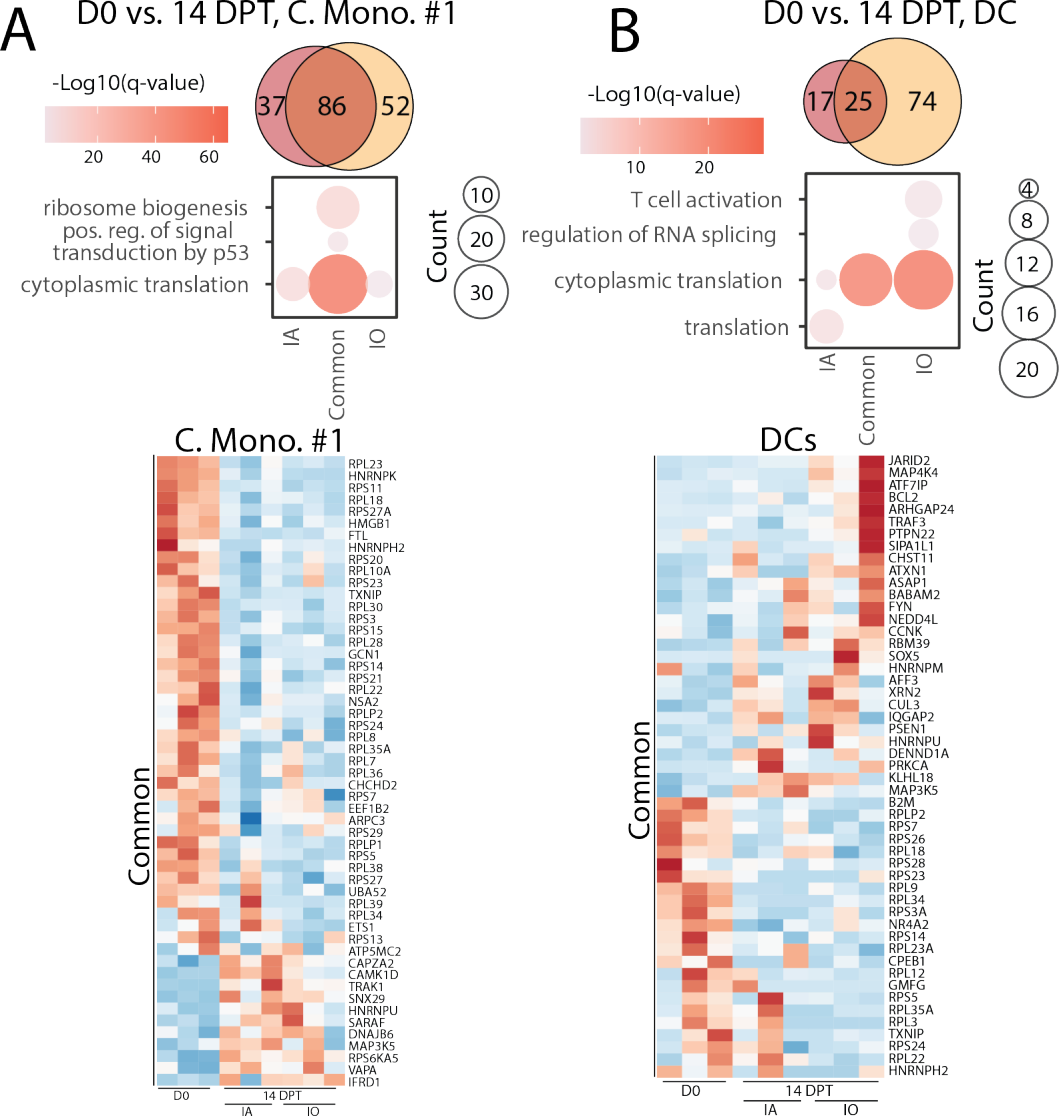
Peripheral immune cells mount a response during chronic disease. Venn diagram and GO terms of unique and common DEGs between the IO and IA animals at 14 DPT within (**A**) monocyte cluster #1 and (**B**) DCs.

At 14 DPT, the frequency of classical monocyte population #1 (C.Mono.#1) increased significantly in IO animals, while that of C.Mono.#2 population decreased significantly in both IO and IA groups. The frequency of CD4 T cells was decreased in the IA group at 14 DPT relative to 0 and 28 DPI (Fig. 4C). Finally, the frequency of CD8 T cells was reduced at 14 DPT in both IA and IO groups (Fig. 4C). We focused on monocytes and DCs due to their extensive transcriptional differences observed at 28 DPI, as well as B cells due to the increases in immunoglobulin, CXCL13, and proliferating B cells in the periphery (Table S1; Fig. 6).

A large overlap in DEGs was noted between the IO and IA groups at 14 DPT for both monocytes and DCs. Most DEGs were enriched in GO terms related to translation (Fig. 6A and B). However, there were differences between the IO and IA groups. *CAPZA2, CAMK1D, TRAK1, SNX29, HNRPU,* and *SARAF*, which are involved in cytoskeletal dynamics, calcium signaling, intracellular transport, and gene expression, were uniquely increased in the IO group. Several *RPS* and *RPL* genes, which encode ribosomal proteins, were uniquely upregulated in the IA group (Fig. 6A). Similarly, DEGs that are unique to DCs from IA animals mapped to translation (*RPL* and *RPS* genes), while those unique to the IO group play a role in RNA splicing (*RBM39* and *XRN2*) and T cell activation (*TRAF3* and *FYN*) (Fig. 6B).

The majority of the DEGs detected 14 DPT in the B cell subset were shared between the two experimental groups. Shared DEGs in subset #1 were primarily involved in RNA splicing and regulation of protein stability (*HNRNPH2* and *HSP90AA1*), survival and proliferation (*JUND* and *MAPK1*), mitochondrial activity (*ATP5MC2*), and translation (*RPL* and *RPS* genes) (Fig. S3A). DEGs unique to IO animals were enriched in actin filament-based processes (*ARHGAP26* and *DOCK2*), while those unique to IA animals were uniquely enriched in viral release from host cells (*IST1* and *RAB7A*) (Fig. S3A). Similarly, shared DEGs in B cell subset #2 were enriched predominantly in cytoplasmic translation and inflammatory processes (*COX2, MAPK1, CORO1A,* and *MAMU*). DEGs unique to IA animals played a role in the formation of cytoplasmic granules (*DDX3X* and *CELF1*) and ubiquitination (*FBXO33* and *ARIH1*). DEGs unique to IO animals mapped to hormonal response (*NCOA3*), host defense (*MAMU-DRB1* and *FOS*), epigenetic changes (*ESCO1, TET2,* and *KDM4C*), and cellular growth (*RPS6KB1* and *CRIP1*) (Fig. S3B).

### Bb infection induces mononuclear myocarditis

**Fig 7:**
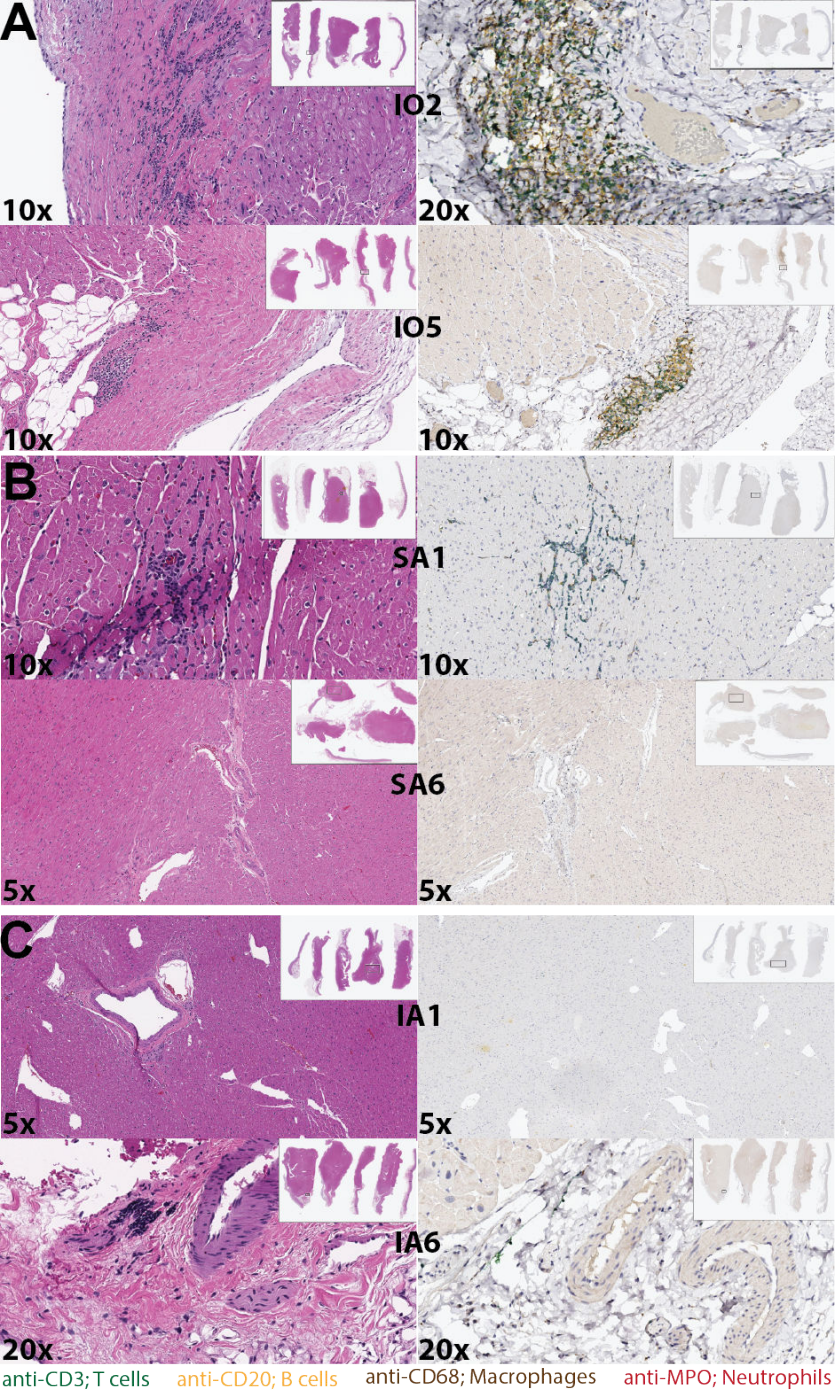
Doxycycline prevents mononuclear myocarditis. Representative H&E with their corresponding immunohistochemistry images at the indicated magnification of heart tissue from (**A**) IO, (**B**) SA, or (**C**) IA. 4′,6-Diamidino-2-phenylindole (blue) was used as a general DNA stain; anti-CD20 was used to stain for B cells (yellow), anti-CD3 for T cells (green), anti-CD68 for macrophages (brown), and anti-MPO for neutrophils (red).

*Borrelia* can disseminate into joints, the nervous system, and heart tissue. Thus, hematoxylin and eosin (H&E) staining was performed at necropsy on the joints, several areas of the central nervous system (CNS), and heart tissue to investigate immune cell migration. Histology from the joints and CNS was unremarkable (Fig. S4). In contrast, H&E of the heart tissue revealed mononuclear myocarditis in all animals within the IO group (Fig. 7A) and one animal in each of the SA and IA groups (SA1 and IA6, Fig. 7B and C). Immunohistochemistry staining revealed the presence of T cells, B cells, and macrophage infiltration throughout the heart tissue of the IO animals (Fig. 7A; Fig. S5A), while antibiotic-treated animals had minimal infiltration (Fig. S5B and C). Given the myocarditis, we next investigated whether *Borrelia* could be detected in the heart tissues. Low levels of *Borrelia* DNA were detected in three out of six animals by real-time quantitative PCR (qPCR) (Fig. S5D). However, we were not able to detect spirochetes by indirect immunofluorescence analysis (IFA) (data not shown). This may be due to the time of collection at necropsy (120 or 157 DPI).

### Bb infection and doxycycline treatment induce loss of butyrate-producing bacteria

**Fig 8:**
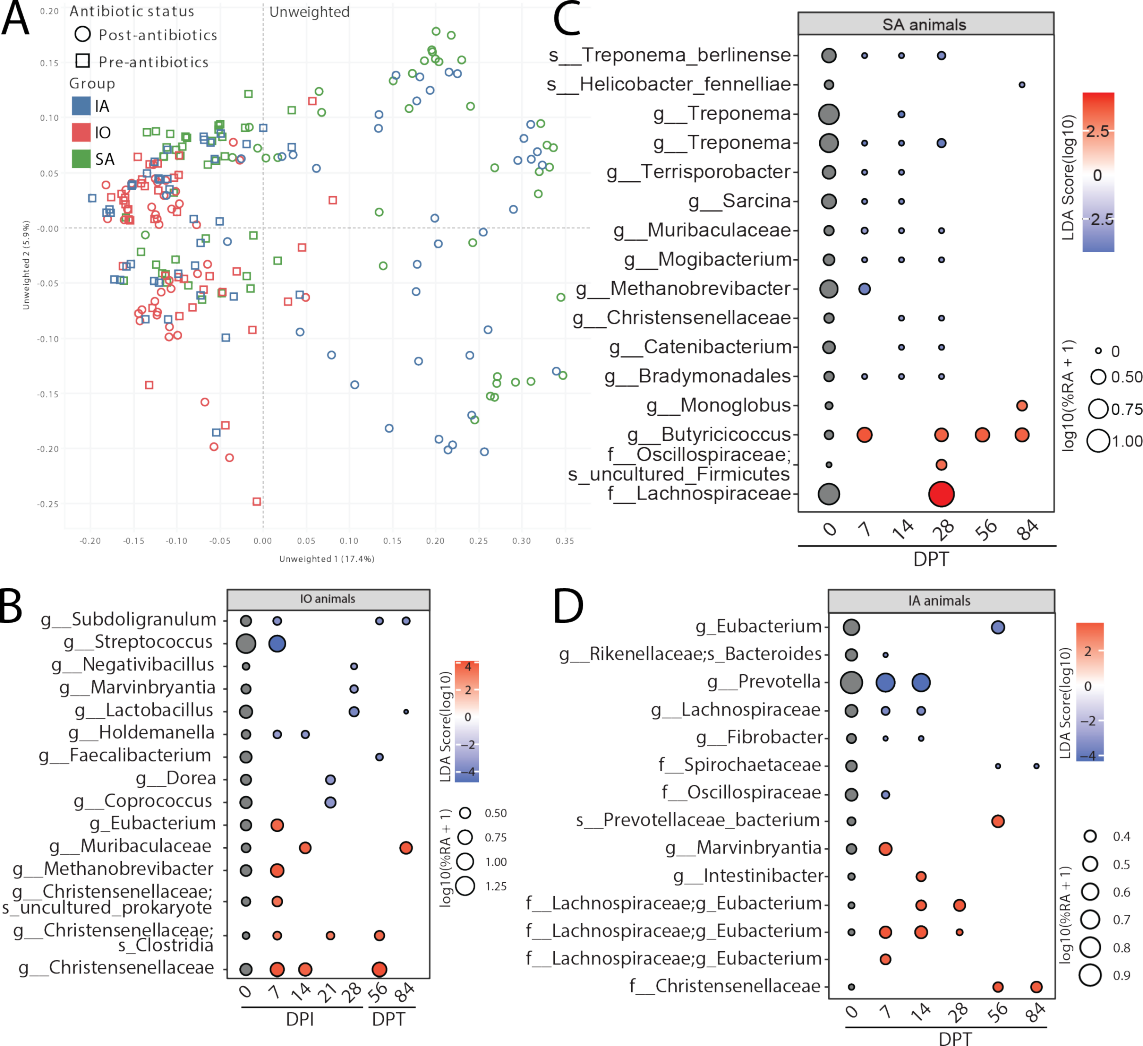
Microbiome profiling reveals loss of short-chain fatty acid-producing commensals. (**A**) The unweighted principal coordinate analysis plot of the fecal swabs at the indicated DPI. Bubble plots derived from LEfSe analysis of the microbiome profiling of the feces in (**B**) infected animals without doxycycline treatment at 0–28 DPI, 56 DPT, and 84 DPT, (**C**) SA controls at 0–84 DPT (after doxycycline treatment), and (**D**) IA animals at 0–84 DPT. The size of each bubble represents the percent relative abundance, and color represents the linear discriminant analysis (LDA) score.

We next investigated the effects of Bb infection and antibiotic treatment on the gut microbiome via longitudinal 16S rRNA sequencing of fecal samples. The unweighted principal coordinate analysis (PCoA; presence or absence of species) showed a separation of antibiotic-treated animals’ microbiomes compared to untreated (Fig. 8A), while the weighted PCoA (relative abundance of species) did not show clear differences (Fig. S6A). We carried out linear discriminant effect size (LEfSe) analysis to uncover changes (Fig. 8; Fig. S6). Longitudinal changes before doxycycline treatment within the SA group (0–28 DPI) were used to control for changes in fecal microbiome associated with sedation and daily fluctuations (Fig. S6B). Bb infection decreased the relative abundance of bacteria that are major commensals of the macaque gut (*Streptococcus*), produce butyrate (*Subdologranulum*, *Marvinbryantia*, *Faecalibacterium*, and *Coprococcus*), promote mucus secretion (*Lactobacillus*), regulate host metabolism (*Holdemanella*), and ferment sugars (*Dorea*). Interestingly, the prevalence of other species that produce non-butyrate short-chain fatty acids (Muribaculaceae), remove hydrogen gas from fermentation (*Methanobrevibacter*), and help establish other microbial taxa (Christensenellaceae) was increased during Bb infection (Fig. 8B). These data suggest a potential compensatory mechanism. However, the loss of butyrate producers, which are integral for maintaining a healthy gut lining (30–32), does not appear to be compensated.

Next, we investigated the impact of doxycycline treatment (SA group) on the gut microbiome (Fig. 8C). This broad-spectrum antibiotic reduced the abundance of commensals (*Treponema*, *Helicobacter*, and *Mogibacterium*), fatty acid and short-chain fatty acid producers (*Terrosporobacter*, *Muribacterium*, and *Catenibacterium*), methanogens (*Methanobrevibacter*), and fermenters (Christensenellaceae). Monoglobus, uniquely increased due to doxycycline (Fig. 8C), is associated with neutrophilic inflammation and liver injury (33). Infected animals treated with doxycycline (IA group) showed decreased abundance of species involved in fermentation (Rikenellaceae), and degradation of amino acid, carbohydrate, fiber, and cellulose (*Prevotella*, *Fibrobacter*, and Spirochaetaceae). Once again, the abundance of other species associated with short-chain fatty acid production (Prevotellaceae and Marvinbryantia) and fermentation (Christensenellaceae) was increased in the IA group, potentially to compensate for the initial loss (Fig. 8D). Despite these shifts in microbial community composition, the overall gut diversity as measured by amplicon sequence variants remained unchanged throughout the study except for a modest increase in diversity at 21 DPI in the IO group (Fig. S6C).

## DISCUSSION

This study details the longitudinal immune response and gut microbial perturbations caused by doxycycline-treated and untreated Bb infection. We were unable to detect Bb by qPCR in the skin at the injection site, in the blood, or the CSF. While other groups have previously reported Bb detection by qPCR at sites of injection in macaques (24), these results were obtained after a total inoculation dose of 3.2 × 10^8^ spirochetes, with 10^7^–10^8^ spirochetes injected in each location. In contrast, our model used a total inoculum of 10^4^ spirochetes divided between six locations in the dorsum to more closely recapitulate physiological bacterial burden after a tick bite (34). The inability to detect Bb by PCR is a major challenge for the field (6–8). One potential explanation is the ability of *Borrelia* to quickly migrate from the skin to other anatomical sites such as the joints, central nervous system, and heart (27, 28). Our inability to detect Bb in synovium, the CSF, and only sporadically in the heart is most likely due to the time point at which samples were harvested (120 and 157 DPI). Regardless, seroconversion and transcriptional changes at the site of injection were indicative of a robust antimicrobial response following Bb inoculation.

Untreated infected animals produced Bb-specific immunoglobulins at uncharacteristically slow rates compared to other pathogens (35), which is a common challenge for Lyme serological diagnostics (9–13). This delayed immunoglobulin production may be due to the small inoculum used (36) and *Borrelia*’s ability to evade the immune system (14–17). Doxycycline treatment led to a rapid reduction in IgM and IgG levels, consistent with a reduction of antigen load after treatment.

We detected a significant increase in circulating levels of CXCL13, a B cell chemoattractant, which plummeted after doxycycline treatment. CXCL13 was also detected in the cerebrospinal fluid of untreated macaques. This chemokine has previously been used as a biomarker for Lyme neuroborreliosis (37, 38). Detection of this chemokine is potentially linked to the use of strain 297, which is a clinical isolate from cerebrospinal fluid (39). The precipitous drop in CXCL13 after doxycycline treatment may be due to its immunosuppressive effects (40–42) and inhibition of B cell functions (43) along with the bactericidal effects. In concordance with the lack of changes in immune mediators in the blood, we saw minimal changes in myeloid or lymphoid cell frequencies in circulation. Only a modest increase in proliferating B cells was detected in infected animals, despite the prolonged high levels of CXCL13 in these animals.

In corroboration with our flow cytometry and Luminex data suggesting a slow and limited immune response, there were minimal gene expression changes within circulating immune cells. Minor gene expression changes within monocytes and DCs indicate a blunted inflammatory response in accord with the known immune evasion properties of Bb (14–17). During the later stages of disease, DEGs in monocytes, dendritic cells, and B cells were strongly enriched in cytoplasmic translation, an essential process for protein production, which corresponded to the increase in antibody detection in the periphery. DCs from IA animals did not exhibit increased expression of genes associated with T cell activation. It is possible that more robust immune responses would have been detected within draining lymph nodes and tissues rather than the blood. Future studies would benefit from investigating these anatomical locations.

The joints (knees and meniscus) as well as areas of the CNS (cerebellum, dura, trigeminal, and spinal cord) showed no evidence of inflammation. In contrast, mononuclear myocarditis was observed in all the IO animals, which was previously reported in other mouse (44–46) and macaque models (24) of borreliosis. These changes in the heart are in line with common symptoms of Lyme carditis patients such as fatigue and malaise, lightheadedness, chest pain, and heart palpitations (47, 48). Immunohistochemistry of these sections revealed that these immune infiltrates were T cells, B cells, and, to a lesser extent, neutrophils and macrophages. We also observed very low levels of borrelial DNA in half of the IO animals’ heart tissue by qPCR, but no evidence of Bb or its antigens by IFA, which is in contrast to other NHP studies (49). This may be due to the lower inocula used for this study or the sampling time point at necropsy, and the possibility that mononuclear myocarditis is a self-sustaining inflammatory process.

Recent links between gut dysbiosis and common symptoms of LD (20–22) led us to perform 16S rRNA sequencing to examine changes in the gut microbiome during untreated infection and doxycycline treatment. *Borrelia* infection alone caused loss of some gut commensals (50) and SCFA producers, notably those that make butyrate, a key factor in maintaining the gut lining by strengthening the interaction between tight-junction proteins (30–32). Additionally, the relative abundance of bacteria that promote mucus production, aid host metabolism, and ferment sugars and polysaccharides was decreased during infection (51, 52). Other beneficial bacteria were increased, which may be a compensatory mechanism to maintain gut integrity. Doxycycline treatment alone also led to a loss of different species of commensals, SCFA producers, methanogens, and fermenters than Bb infection. The combination of disease and antibiotics resulted in an exacerbated loss of important SCFA producers. We posit that this loss of commensals compromises the gut barrier (53, 54), leading to immune activation and systemic inflammation (55).

This study has some limitations. Animals were examined 3 months after antibiotic treatment, so later-stage symptoms/signs of infection could not be assessed. We used an intradermal inoculation method to infect animals with Bb rather than a tick bite. This was intentional to ensure that each animal received the same inoculum dosage. However, this method does not fully recapitulate a physiological infection due to the absence of tick salivary proteins, which are critical to establish infection (56, 57), as well as the lack of time and physiological cues from tick engorgement for Bb to properly modulate their outer surface protein expression (58–61), since the bacteria were cultured in Barbour-Stoener-Kelly-II (BSK-II) media. Currently, we have only analyzed the peripheral immune response. Future directions would be to more closely investigate the immunological state of cells within the heart or lymph nodes, where robust immune responses may be occurring at necropsy. Finally, we only observed 28 DPI by single-cell RNA sequencing before antibiotics. There may be a key time point of antimicrobial defense in the first 4 weeks that went unobserved.

In summary, the data presented here provide supporting evidence for the role of CXCL13 as a biomarker for borreliosis. Moreover, our observations suggest that B cell activity, mononuclear myocarditis, and gut dysbiosis may contribute to the sequelae of LD. Interventions such as probiotic supplementation in conjunction with antibiotic treatment may be beneficial.

## MATERIALS AND METHODS

### Cohort description, animal infection, treatment, and sample collection

This study was conducted using two Japanese macaque (*Macaca fuscata*) cohorts, each containing three experimental groups for a total of six animals per group: (i) infection + antibiotics; (ii) infection only; and (iii) saline injection + antibiotics. The first group was infected with 10^4^ Bb strain 297 divided across six sites in the dorsum and remained untreated throughout the course of the study (IO). The second group was infected with Bb as described for group (i), then subsequently treated with 2 mg/kg of body weight/day twice daily of doxycycline for 21 days (62) starting at 28 or 42 days post-infection (*n* = 3 each; IA). Doxycycline was administered at these times to mimic the average length between the tick bite and when symptoms and treatment begin (63). The last group was injected with an equal volume of saline and administered doxycycline (SA) as described for group (ii). We collected feces and blood at −14, 0, 3, 7, 14, 21, and 28 DPI as well as 7, 14, 21, 28, 35, 42, 56, 70, 84, 98, and 112 DPT with doxycycline. Skin biopsies at the injection sites were collected at 3 and 7 DPI. We harvested feces, blood, and heart tissue at necropsy.

Female Japanese macaques between 4 and 15 years of age at study initiation were housed at the ONPRC. All macaques in this study were managed according to the ONPRC animal care program, which is fully accredited by AAALAC International and is based on the laws, regulations, and guidelines set forth by the United States Department of Agriculture (e.g., the Animal Welfare Act and Animal Welfare Regulations, the Guide for the Care and Use of Laboratory Animals, 8th edition [Institute for Laboratory Animal Research]) and the Public Health Service Policy on Humane Care and Use of Laboratory Animals. Animals received *ad libitum* access to food (Purina 5000 Fiber-balanced Monkey Diet, Purina Mills, Richmond, IN, USA) and fresh water. The nutritional plan utilized by the ONPRC is based on National Research Council recommendations and supplemented with a variety of fruits, vegetables, and other edible items as part of the environmental enrichment program established by the Behavioral Services Unit. All diets and dietary enrichment were standardized throughout the study. Animals within the same experimental groups were socially housed when possible.

### *Borrelia burgdorferi* strain 297 stock, culture, and quantitation

Strain 297 was originally isolated from human CSF (39). It was provided to our group by Dr. Catherine Brissette (University of North Dakota) within two passages after reisolation from infected mice (64). The bacteria were grown in BSK-II medium supplemented with 6% rabbit serum at 35°C to the mid-exponential phase (~10^7^ bacteria/mL) (65, 66). Cultures were counted using a Petroff-Hauser counting chamber with dark field microscopy, centrifuged at 8,000 × *g* for 30 minutes at 4°C, washed with PBS twice, and resuspended at 10^4^ spirochetes/mL before being inoculated.

For real-time qPCR bacterial quantification, DNA was extracted from skin biopsies, CSF, serum, and heart tissue using the DNeasy PowerSoil Pro Kit (Qiagen, Germantown, MD, USA) according to the manufacturer’s instructions. Bb burden was determined by qPCR using SsoAdvanced Universal SYBR Green Supermix (Bio-Rad, Hercules, CA, USA) and primers specific for the *flaB* gene of *Borrelia* species (64). Each run was initiated at 50°C for 2 minutes, then at 95°C for 10 minutes, followed by 40 cycles at 95°C for 15 s, then 60°C for 1 minutes using a QuantStudio 3 Real-Time PCR System (Thermo Fisher Scientific). Purified 297 genomic DNA was used as the quantification standard.

### ELISA

IgM and IgG antibody titers against Bb were determined using enzyme-linked immunosorbent assay. Plates were coated with 1 mg/mL Bb 297 bacterial lysate overnight at 4°C. The plates were washed three times with 0.05% Tween-PBS. Heat-inactivated (56°C for 30 minutes) plasma samples were then added in threefold dilutions in duplicates for 1.5 h at room temperature. After washing with 0.05% Tween-PBS, Goat anti-monkey IgM or IgG (Fc) HRP (Brookwood Biomedical, Jemison, AL, USA) was diluted and then added to each well and incubated for 1–1.5 h. The plates were then washed three times with 0.05% Tween-PBS and incubated for 20 minutes in a solution containing o-phenylenediamine·2HCl substrate (Sigma-Aldrich, St. Louis, MO, USA) diluted in substrate buffer and 30% H_2_O_2_. The reaction was stopped by adding 1 M HCl to each well. Absorbance at 490 nm was measured using the SpectraMax iD3 (Molecular Devices, San Jose, CA, USA) plate reader. Each plate included a positive control that was used to normalize the data. Basal absorbance was observed to be approximately 10^2^ AU at 0 DPI and throughout the study in the SA group.

### Flow cytometry

Peripheral blood mononuclear cells (10^6^ PBMCs) were surface stained with antibodies against CD4, CD20, CD27, IgD (Biolegend, San Diego, CA, USA), CCR7 (BD Biosciences, Franklin Lakes, NJ, USA), CD8b (Beckman Coulter, Brea, CA, USA), and CD28 (Tonbo Biosciences, San Diego, CA, USA). Cells were then fixed and permeabilized before the addition of anti-Ki67 (BD Biosciences, Franklin Lakes, NJ, USA). B cells (CD20) and T cells (CD8b and CD4), as well as naive and memory subsets, were identified as previously described (67). BAL cells (10^6^) were stained with CD8a, CD14, CD16, CD123, CD206, HLA-DR (Biolegend, San Diego, CA, USA), CD11c (Invitrogen, Waltham, MA, USA), and Granzyme-B (BD Biosciences) to assess the frequency of innate immune cells (67).

### Luminex

The levels of immune mediators in plasma were analyzed using the R&D 36-plex NHP XL Cytokine Premixed Kit (Bio-Techne, Minneapolis, MN, USA) for chemokines (CCL2, CCL5, CCL11, CCL20, CXCL2, IL-8/CXCL8, CXCL10, CXCL11, and CXCL13), pro- and anti-inflammatory cytokines (IFNα, IFNβ, IFNγ, IL-1β, IL-5, IL-6, IL-12 p70, IL-2, IL-4, IL-7, IL-10, IL-13, IL-15, IL-17, and IL-21), as well as growth factors (TGF-β, BDNF, FGF, G-CSF, PDGF-AA, PDGF-BB, and VEGF). Samples were acquired using the MAGPIX xMAP (Luminex Corporation, Austin, TX, USA). Data were analyzed using the Luminex Xponent software with an eight-point logistic regression curve.

### Immunohistochemistry analysis

Immunohistochemistry staining was done on the Ventana Discovery Ultra platform. FFPE sections (4 µm) were cut and mounted onto plus charged slides that were dried overnight at 60°C. Deparaffinization was done before antigen retrieval with Ventana CC1 buffer for 48 minutes. Myeloperoxidase (predilute, Roche 05267692001) was added at 37°C for 32 minutes. Post-primary peroxidase quench was done with Discovery Inhibitor (Ventana 760-4840) for 4 minutes at 37°C. Slides were incubated with Discovery Ultramap anti-rabbit-AP (Ventana 760-4314) for 12 minutes at 37°C, followed by Discovery Red Chromogen Kit (Ventana 760-228) for 4 minutes at 37°C, CD68 KP1 (predilute, Dako IR609) for 60 minutes at 37°C, anti-mouse HQ (Ventana 760-4814) for 20 minutes at 37°C, anti-HQ-HRP for 20 minutes at 37°C, and Discovery Teal HRP Kit (Ventana 760-247) for 12 minutes. Antibody denaturation was done with Ventana CC2 (Ventana 950-223) at 100°C.

Slides were then incubated with Dako Dual (AP/HRP) Endogenous Block (Agilent K4065) for 16 minutes at 37°C followed by CD3 2GV6 (Ventana 790-4341), Omni-map anti-rabbit-HRP (Ventana 760-4311), DAB (Ventana 760-159), CD20 (Ventana 760-2531), Ultramap anti-mouse-AP (Ventana 760-4312), and Discovery Yellow (Ventana 760-239), all at 37°C for 20 minutes. Counterstain was done with Mayer’s hematoxylin for 5 minutes. Slides were air-dried overnight before permanent mounting. All runs included a human tonsil as control for staining. Indirect immunofluorescence analyses for Bb antigen in heart tissue were done as described in reference 49 with rabbit polyclonal anti-Bb primary antibody (68) and goat anti-rabbit IgG secondary antibody (Thermo Fisher, A-11034) that was supplied to us by the Embers laboratory.

### 16s amplicon sequencing and bioinformatics analysis

Amplification of the hypervariable V4 region of the 16s rRNA gene was performed using the 515F/806R PCR primers and analyzed as previously described (69). The forward primers were conjugated with a 12 bp barcode (70). Each reaction was run in duplicate and prepared with GoTaq master mix (Promega Corporation, Madison, WI, USA). Cycling conditions were 94°C for 3 minutes, 37 cycles at 94°C for 45 s, 50°C for 1 minute, and 72°C for 1 minute, followed by a final cycle at 72°C for 10 minutes. The PCR products were multiplexed using Quant-iT PicoGreen dsDNA Assay Kits and dsDNA Reagents (Thermo Fisher). The resulting library was spiked with ~15%–20% PhiX and sequenced on an Illumina MiSeq. Raw FASTQ 16s rRNA gene amplicon sequences were processed using the QIIME2 analysis pipeline (71). Sequences were demultiplexed and filtered using DADA2 (72). MAFFT was used to align the sequence variants, while FastTree2 was utilized to construct a phylogenetic tree (73, 74). Taxonomy was assigned to sequence variants using q2-feature-classifier against the SILVA database (release 138) (75). The samples were rarified to 10,000 sequences per sample. QIIME2 was also used to generate alpha diversity metrics, while beta diversity was estimated using weighted and unweighted UniFrac distances (76). Differentially abundant bacteria between groups were identified via the LEfSe algorithm with a linear discriminant analysis score cutoff of 2 (77).

### Bulk RNA library generation and bioinformatics analysis

Frozen skin biopsies at 3 and 7 DPI were thawed and homogenized in QIAzol lysis reagent (Qiagen) with the Mini BeadBeater 96 Disruptor (BioSpec Products, Bartleville, OK, USA) and 1.4 mm ceramic beads (Omni International, Kennesaw, GA, USA). RNA was extracted using the Qiagen RNeasy Mini Kit (Qiagen) as per the manufacturer’s instructions. Bulk RNA libraries were generated from the RNA extract with the NEBNext Ultra II RNA Kit (New England Biolabs, Ipswich, MA, USA). The libraries were multiplexed and sequenced to a depth of 20 million reads. Reads were assessed using FASTQC, followed by trimming and aligning the sequences to the *Macaca mulatta* Mmul10 genome since the *Macaca fuscata* genome has not been annotated. DEGs were identified and analyzed longitudinally using the Short Time-series Expression Miner and the R package microarray Significant Profiles (maSigPro). Functional enrichment of DEGs was carried out using Metascape.

### Single-cell RNA sequencing library generation and bioinformatics analysis

Cryopreserved PBMCs at 0 and 14 DPI, as well as 14 DPT, were thawed and then stained with propidium iodine (BioLegend; San Diego, CA, USA) to sort viable cells using an iCyt-Sony Cell Sorter System. Live cells were counted on a LUNA-FL Dual Fluorescence Cell Counter (Logos Biosystems, South Korea), tagged with cell multiplexing oligos, and pooled. Single-cell RNA libraries were prepared and analyzed as previously described (69). The cells within each pool were diluted to a concentration of 1,600 cells/µL in ice-cold PBS with 0.04% BSA. Single-cell suspensions were loaded on the 10X Genomics Chromium Controller with a loading target of 20,000 cells. The libraries were prepared using the Chromium Single Cell 3′ Feature Barcoding Library Kit with the v3.1 chemistry (10X Genomics, Pleasanton, CA, USA). Libraries were sequenced using an Illumina NovaSeq with a target of 30,000 reads per cell RNA library and 2,000 reads per cell hashtag-oligo barcode library.

The reads were aligned and quantified using the Cell Ranger Single-Cell Software Suite (version 4.0, 10X Genomics) against the Mmul_8 rhesus macaque reference genome using the STAR aligner. Seurat (version 4.1.1) was used for downstream analysis of the aligned reads. The libraries were merged, and cells with ambient RNA (<200 feature counts) and dying cells (>20% total mitochondrial gene expression) were filtered out (78). The resulting multiplexed library was further processed by performing variance stabilization with the SCTransform function, followed by data normalization using NormalizeData. The data were then scaled using ScaleData in preparation for PCA generation, which was accomplished using RunPCA. Thirty principal components were used to cluster the cells before uniform manifold approximation and projection (UMAP) generation using the FindNeighbors and FindClusters (resolution = 0.5) function in Seurat. UMAP generation was accomplished using runUMAP. Cell types were assigned to individual clusters using the FindMarkers function with a log2 fold change cutoff of 0.4. Differential expression analysis was performed using MAST with default settings in Seurat. All comparisons were performed between day 0 and days of notable DPI (8, 14, and 86). Gene expression changes were considered significant with a log fold change ≥ 0.58 and *P* ≤ 0.05.

### Statistical analysis

Statistical analysis was performed using GraphPad Prism software (GraphPad Software Inc., La Jolla, CA, USA). Statistical significance for comparisons involving three or more groups was determined using a two-way ANOVA or a Kruskal-Wallis test with the Benjamini and Hochberg false discovery rate correction method for multiple comparisons. Significance when comparing two groups was measured using a two-tailed, unpaired parametric Welch’s or Student’s *t*-test.

## Data Availability

Data underlying the bulk RNA sequencing (PRJNA1247577), single-cell RNA sequencing (PRJNA1247570), and 16S sequencing (PRJNA1223856) analyses are available at https://www.ncbi.nlm.nih.gov/bioproject/PRJNA1247577, https://www.ncbi.nlm.nih.gov/bioproject/PRJNA1247570, and https://www.ncbi.nlm.nih.gov/bioproject/PRJNA1223856, respectively, within the Sequence Read Archive at NCBI.

## SUPPLEMENTAL MATERIAL

Fig. S1 mbio.01437-25-s0001.png Bulk RNA sequencing of skin biopsies.

Fig. S2 mbio.01437-25-s0002.png Flow cytometry gating strategy and data.

Fig. S3 mbio.01437-25-s0003.png Single cell RNA sequencing of B cells.

Fig. S4 mbio.01437-25-s0004.png H&E stains of locations in the CNS and joints.

Fig. S5 mbio.01437-25-s0005.png Additional histology of heart tissue.

Fig. S6 mbio.01437-25-s0006.png 16S rRNA sequencing of feces.

Table S1 mbio.01437-25-s0007.xlsx Number of DEGs found in each comparison of the single cell RNA sequencing analysis.

ASM does not own the copyrights to Supplemental Material that may be linked to, or accessed through, an article. The authors have granted ASM a non-exclusive, world-wide license to publish the Supplemental Material files. Please contact the corresponding author directly for reuse.
