# Supplementary figures and images for: Borreliosis and doxycycline treatment disrupt gut microbiota and immune responses in nonhuman primates
Source: mBio. 2025 Jun 27;16(8):e01437-25. doi: 10.1128/mbio.01437-25 (PMC12345261; doi:10.1128/mbio.01437-25)

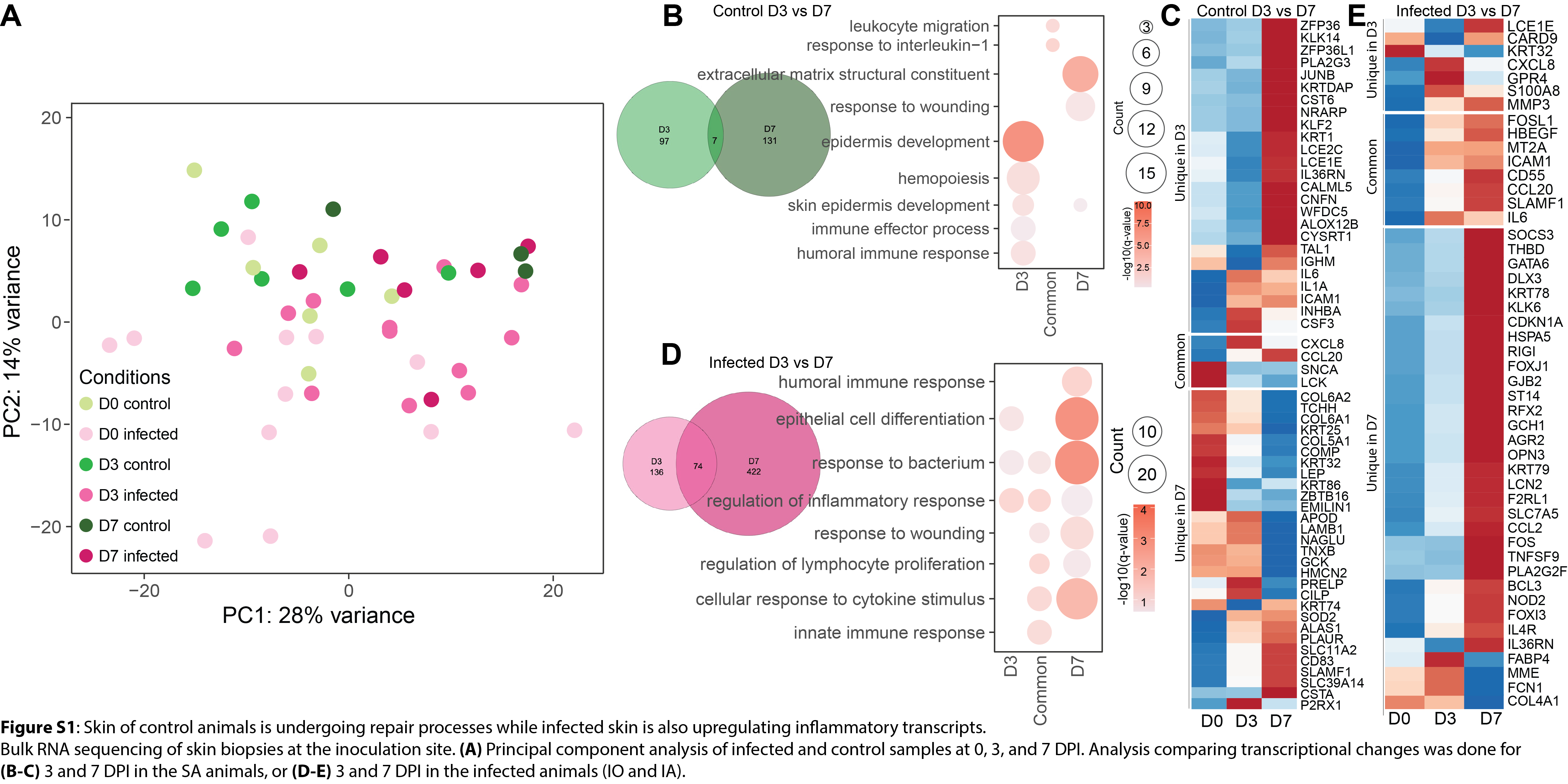

Supplement: Fig. S1 — Bulk RNA sequencing of skin biopsies. [file mbio.01437-25-s0001.png]

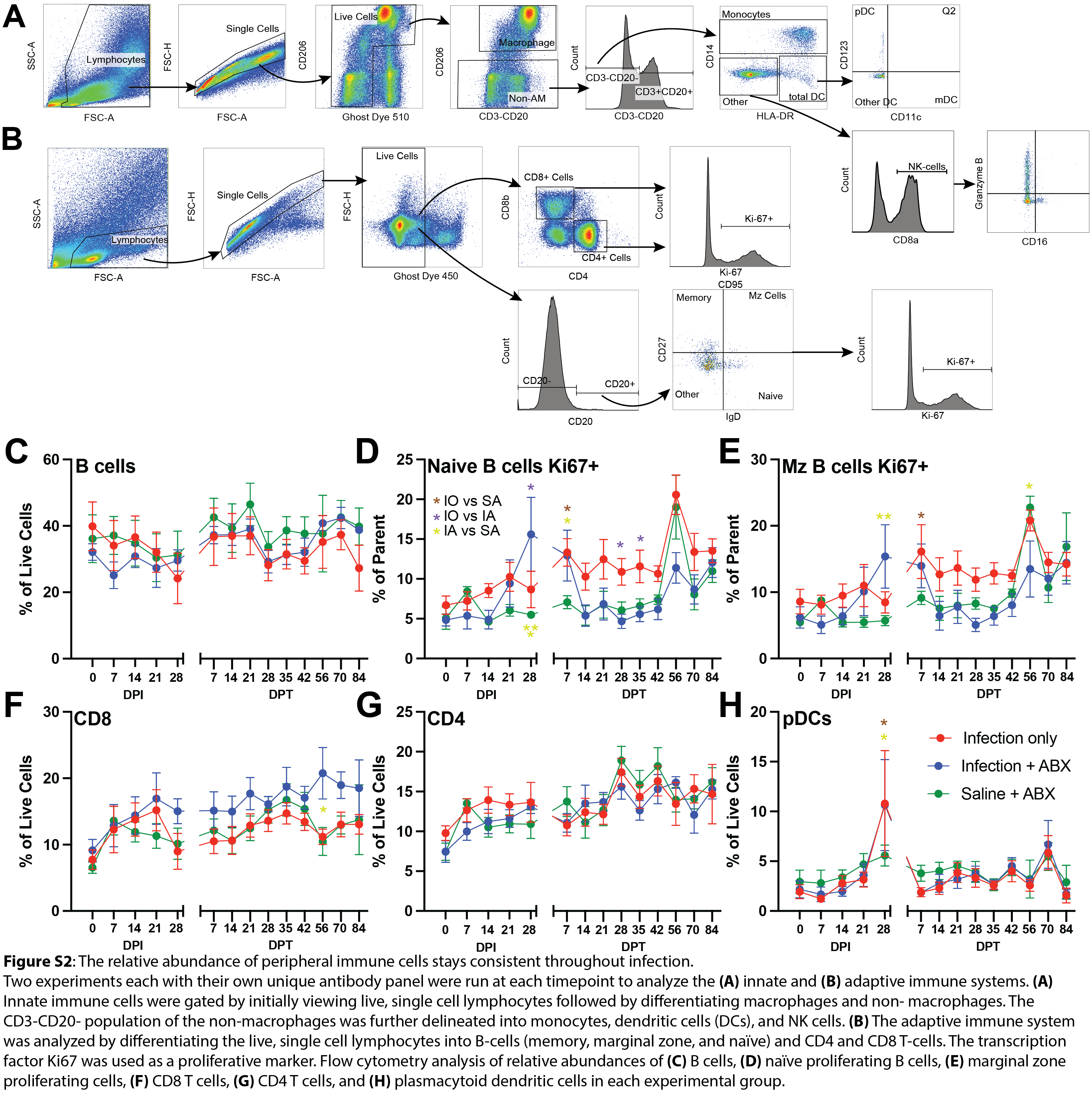

Supplement: Fig. S2 — Flow cytometry gating strategy and data. [file mbio.01437-25-s0002.png]

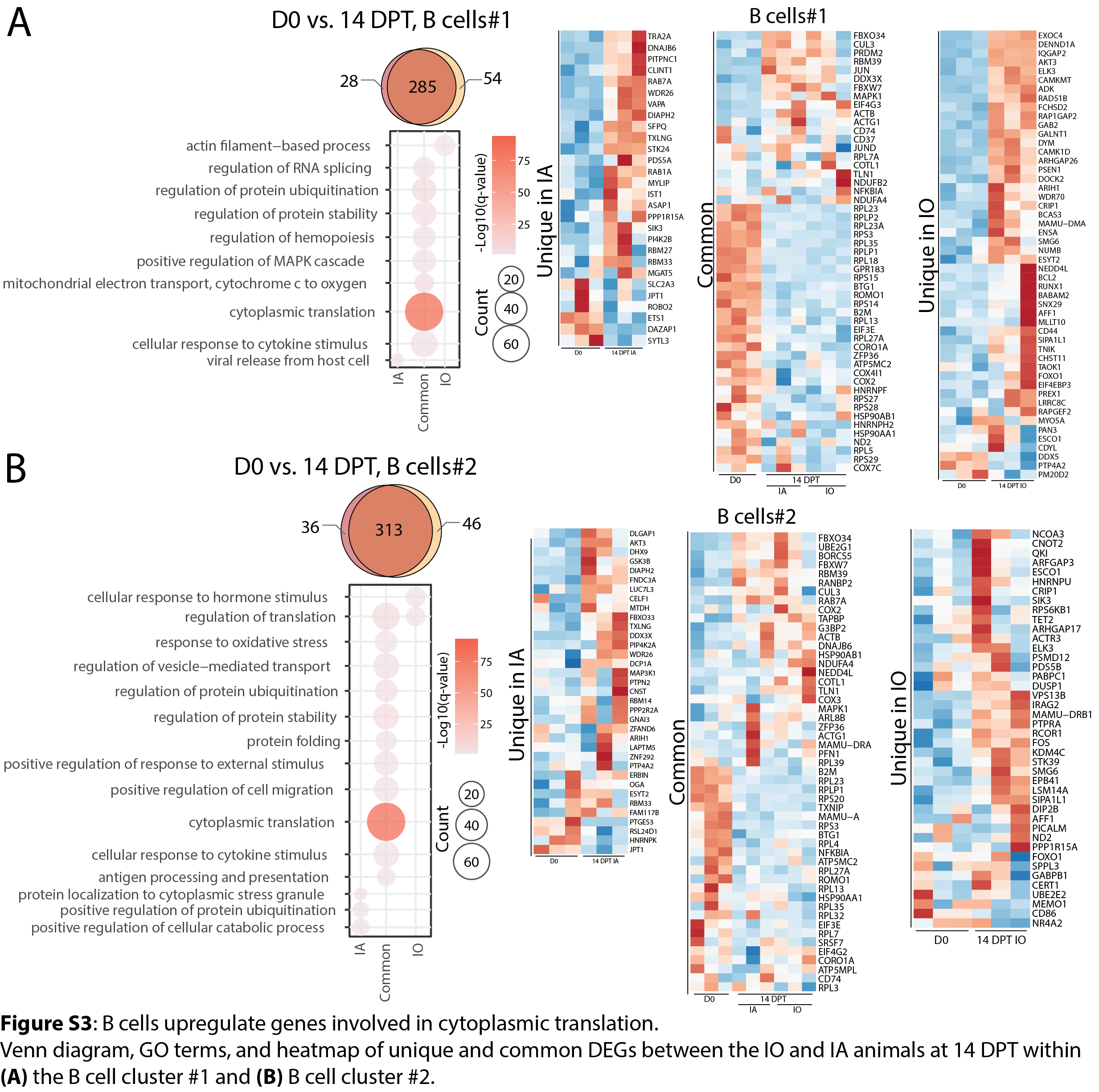

Supplement: Fig. S3 — Single cell RNA sequencing of B cells. [file mbio.01437-25-s0003.png]

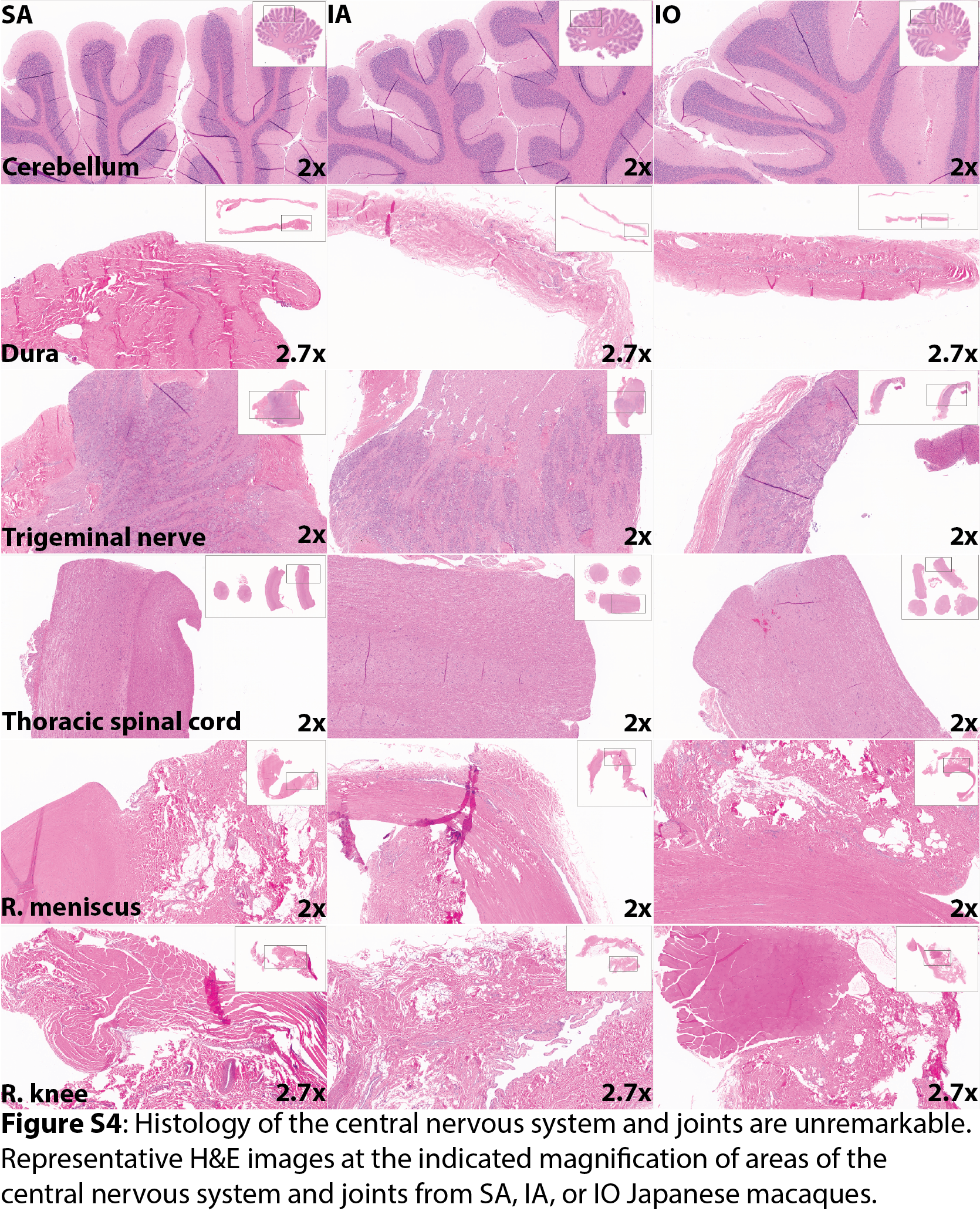

Supplement: Fig. S4 — H&E stains of locations in the CNS and joints. [file mbio.01437-25-s0004.png]

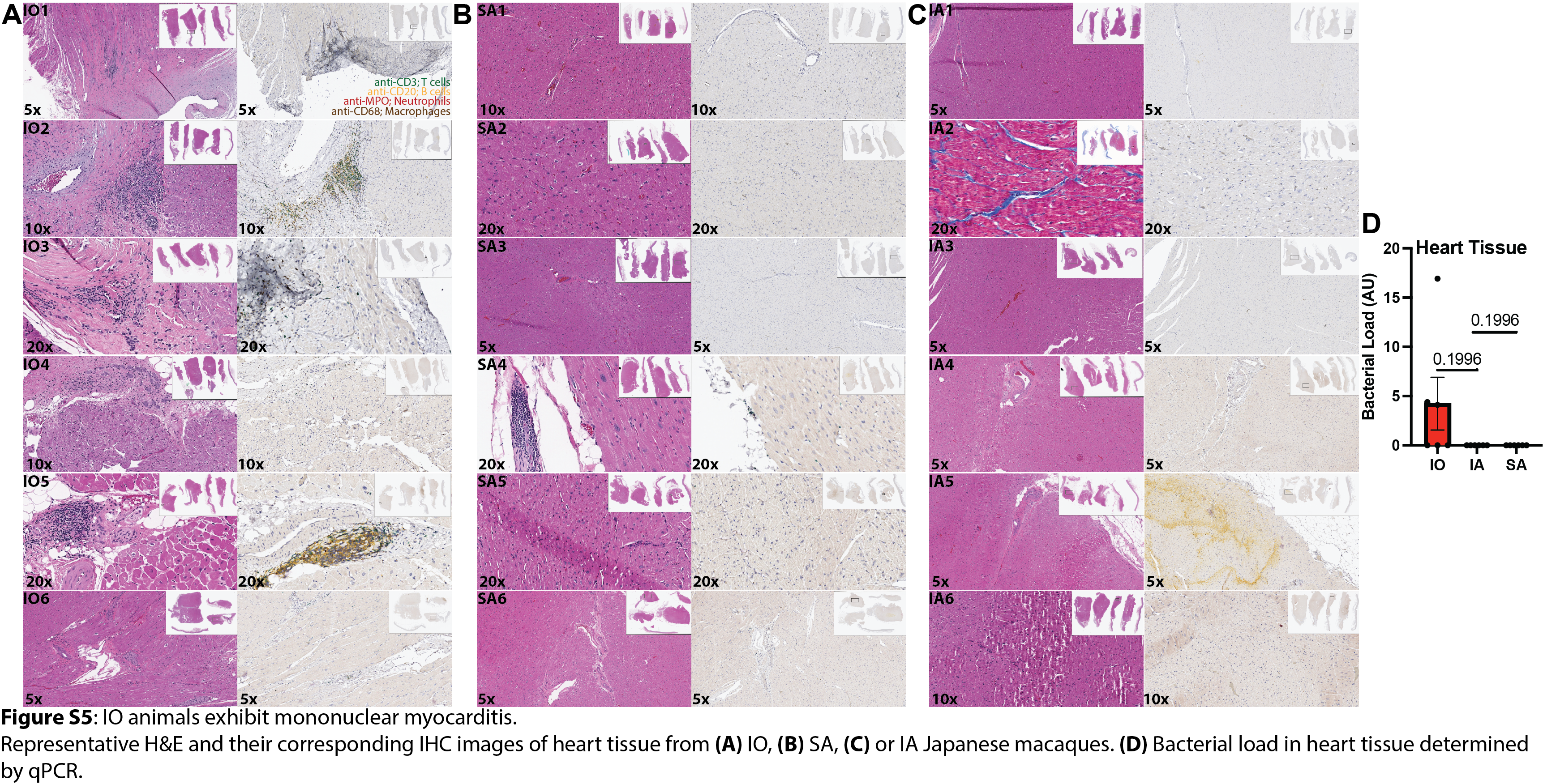

Supplement: Fig. S5 — Additional histology of heart tissue. [file mbio.01437-25-s0005.png]

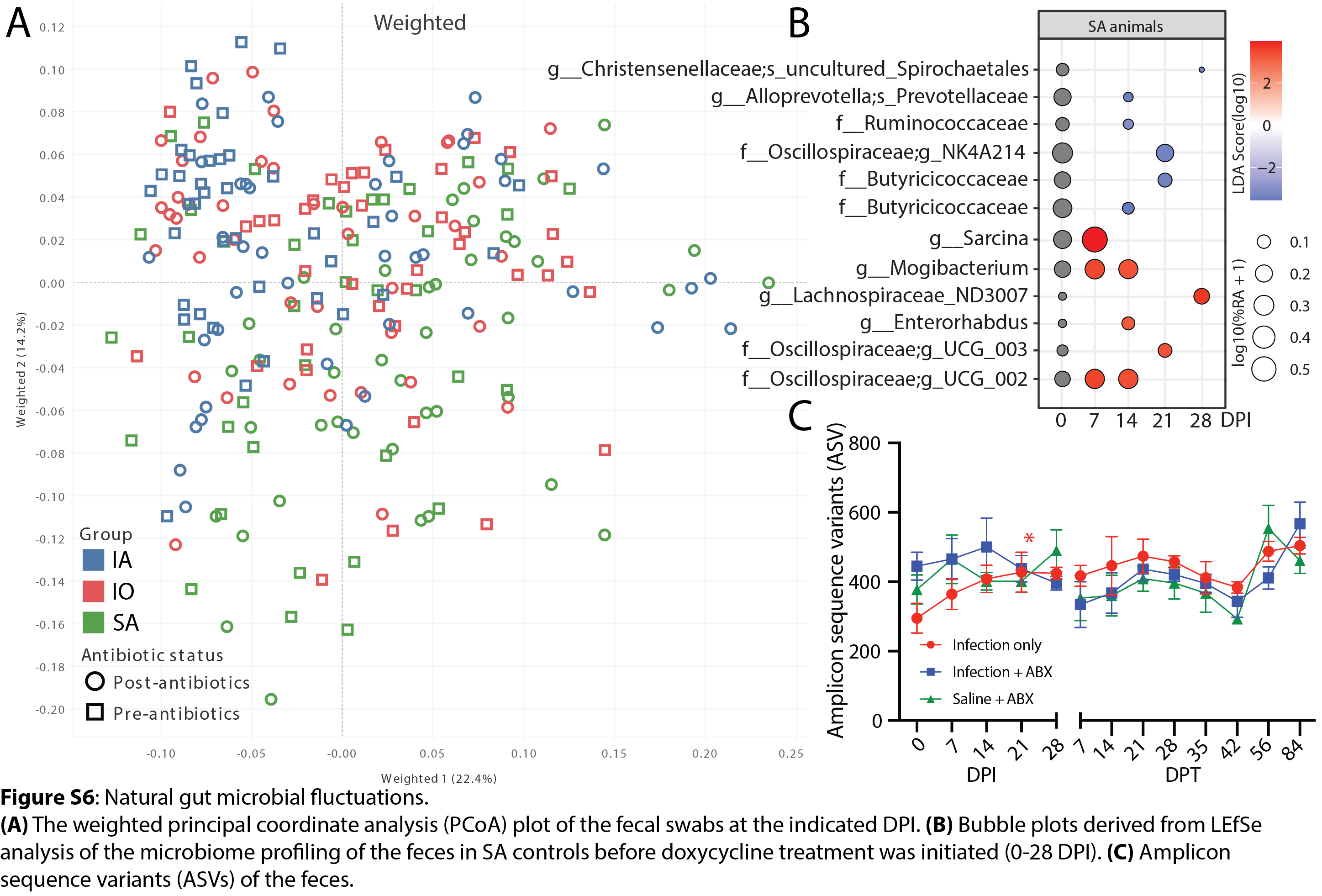

Supplement: Fig. S6 — 16S rRNA sequencing of feces. [file mbio.01437-25-s0006.png]
